# Pilot study for the development of an automatically generated and wearable-based early warning system for the detection of deterioration of hospitalized patients of an acute care hospital

**DOI:** 10.1186/s13690-024-01409-y

**Published:** 2024-10-08

**Authors:** J.J. Reichl, M. Leifke, S. Wehrli, D. Kunz, L. Geissmann, S. Broisch, M. Illien, D. Wellauer, N. von Dach, S. Diener, V. Manser, V. Herren, A. Angerer, S. Hirsch, B. Hölz, J. Eckstein

**Affiliations:** 1 Department of Internal Medicine, University Hospital of Basel, Petersgraben 4, CH-4031 Basel, Switzerland; 2 Innovationmanagement, University of Basel, Basel, Switzerland; 3 School of Life Sciences and Facility Management, Zurich University of Applied Sciences, Research Group Biosensor Analysis and Digital Health, Zurich, Switzerland; 4 Leitwert AG, Zurich, Switzerland; 5 School of Management and Law, Zurich University of Applied Sciences, Head of Management in Health Care, Zurich, Switzerland; 6 School of Life Sciences and Facility Management, Zurich University of Applied Sciences, Research Centre for Computational Health, Zurich, Switzerland

**Keywords:** Early Warning Scores, Continuous monitoring, Wearables

## Abstract

**Background:**

Acute deteriorations of health status are common in hospitalized patients and are often preceded by changes in their vital signs. Events such as heart attacks, death or admission to the intensive care unit can be averted by early detection, therefore so-called Early Warning Scores (EWS) such as the National Early Warning Score 2 (NEWS2), including basic vital parameters such as heart rate, blood pressure, respiratory rate, temperature and level of consciousness, have been developed for a systematic approach. Although studies have shown that EWS have a positive impact on patient outcomes, they are often limited by issues such as calculation errors, time constraints, and a shortage of human resources. Therefore, development of tools for automatic calculation of EWS could help improve quality of EWS calculation and may improve patient outcomes. The aim of this study is to analyze the feasibility of wearable devices for the automatic calculation of NEWS2 compared to conventional calculation using vital signs measured by health care professionals.

**Methods:**

We conducted a prospective trial at a large tertiary hospital in Switzerland. Patients were given a wristband with a photoplethysmogram (PPG) sensor that continuously recorded their heart rate and respiratory rate for 3 consecutive days. Combined with data from the electronic health record (EHR), NEWS2-score was calculated and compared to NEWS2 score calculated from vital parameters in the EHR measured by medical staff. The main objective of our study was to assess the agreement between NEWS2 scores calculated using both methods. This analysis was conducted using Cohen's Kappa and Bland–Altman analysis. Secondary endpoints were compliance concerning the medical device, patient acceptance, data quality analysis and data availability and signal quality for all time stamps needed for accurate calculation.

**Results:**

Of 210 patients enrolled in our study, NEWS2 was calculated in 904 cases, with 191 cases being directly compared to conventional measurements. Thirty-three of these measurements resulted in a NEWS2 ≥ 5, 158 in a NEWS2 < 5. Comparing all 191 measurements, accordance was substantial (K = 0.76) between conventional and automated NEWS2. No adverse effects due to the device were recorded. Patient acceptance was high.

**Conclusions:**

In conclusion, the study found strong agreement between automated and conventional NEWS2 calculations using wearable devices, with high patient acceptance despite some data quality challenges. To maximize the potential of continuous monitoring, further research into fully automated EWS calculations without relying on spot measurements is suggested, as this could provide a reliable alternative to traditional methods.

**Trial registration:**

January 26, 2023, NCT05699967.

**Table:** 

Text box 1. Contributions to the literature
•There is growing evidence that Early Warning Scores (EWS) help to detect deteriorations of health status of hospitalized patients.
•Measurements of EWS are prone to errors due to miscalculation or incomplete recordings, often due to time trouble in clinical practice.
•Therefore, the development of automated EWS calculation systems by Wearables is needed for early detection of possible adverse outcomes.

## Background

Acute deteriorations of patients are often preceded by changes in their vital signs and can thus lead to adverse events in hospital wards, such as admission to the intensive care unit, heart attack or death [1–3]. Such events might be preventable to some degree if deterioration is detected early enough and appropriate measures are taken. Given the increasing workload and complexity of their activities, it can be difficult even for qualified ward staff to observe subtle changes in a patient's state of health and draw the right conclusions.

Systematically monitoring only pre-selected patient groups, as opposed to monitoring all patients in a ward, demands extra attention from nurses, which may lead to errors given the ongoing workload [4]. To address the need for a standardized predictive monitoring, so called Early Warning Scores (EWS) have been developed to systematically assess vital signs of all patients. There are different versions of EWS, but all systems have the same purpose: they are intended to timely identify the risk of patients deteriorating by monitoring the health status of patients during their hospital stay based on routinely measured vital signs by ward staff. When a patient shows signs of deterioration, the EWS triggers a response by the medical staff predefined by an escalation protocol, e.g., an increase of monitoring frequency by ward staff or rapid responses by critical care outreach teams. It is reported that EWS are able to predict an increased risk of mortality or ICU transfer within the next few hours [2]. Current research indicates that the EWS have a positive impact on patient outcomes [5].

The National Early Warning Score 2 (NEWS2) is a well-established EWS that has been used in clinical practice for more than 10 years. It uses respiratory rate (RR), oxygen saturation, need for oxygen therapy, heart rate (HR), blood pressure, level of consciousness and body temperature. A score of 0, 1, 2 or 3 is allocated to each parameter. A higher score means the parameter is further from the normal range. The NEWS2 is then constituted by combining the individual scores of every parameter to an aggregated score, the NEWS2 Score. NEWS2 is a so-called track-and-trigger system. That is, patients are monitored, and a response is triggered when a patient's condition becomes critical. As mentioned above, the triggered response can involve an increased frequency of monitoring by staff or even transfer to a higher level of care, such as an intensive care unit. The process divides patients into critical (alarm is triggered) and non-critical (no alarm). The threshold of a critical health condition is indicated in the NEWS2 with scores > 5.

NEWS2 has been validated for patients older than 16 years of age and its usage in clinical practice has been associated with lower mortality rates and reduced delayed escalations of care [6]. NEWS2 has been updated with a new peripheral oxygen saturation (SpO2) “scale 2” for patients with a prescribed oxygen saturation target of 88–92% to take patients with known hypercapnic respiratory failure into account. The rationale behind this approach was to prevent false alarms triggered by an elevated score, which could lead to an urgent clinical review. In a study by Yu et al. NEWS2 outperformed other commonly used EWS in early identification and prediction of sepsis in the general ward setting [7].

But NEWS2 and other EWS have certain limitations. Typically, they are calculated manually and calculation of the EWS as well as manual data entry is not only time consuming, but also prone to errors and can thus lead to delayed identification of deteriorating patients. Since classical EWS are user-dependent systems, they are prone to incomplete recordings, calculation errors in the EWS and non- adherence to referral protocols [2, 5]. EWS are highly user-dependent and prior studies have shown that in clinical practice, EWS recordings are “frequently incomplete” [8]. The study of van Galen et al. (2016) showed that more than 18% of scores were calculated incorrectly [9]. The main reasons for these failures are a lack of skills and shortage of resources [10]. Respiratory rate, one of the vital signs required for NEWS2, is one of the most important predictors of deterioration, but correctly counting it is associated with considerable additional effort for health care professionals and is prone to errors [1]. Therefore, this vital sign is often neglected in routine vital sign assessment [11].

Considering these factors and aiming to alleviate the workload of healthcare staff, an automated calculation of the Early Warning Score (EWS) could address the mentioned limitations. This would improve both quality of care and patient safety as hospitalized patients at high risk for clinical deterioration could be identified in a timely manner. Mobile sensors (wearables) have the capability to continuously measure and wirelessly transmit data, particularly pertaining to vital signs such as respiratory rate, enabling monitoring of the patient's health status. Wearables can supplement the intermittent recording of some vital signs in the “traditional” way, meaning the usage of stationary devices that are commonly used in hospital wards today. Wearables are therefore cost-effective, flexible and convenient. Furthermore, wearables can also be used for continuous monitoring to close the gap between intermittent measurements of vital signs. The current standard of care is typically around 3 manual measurements per day compared to wearables which can measure continuously (up to several times per second).

The primary goal of this pilot study is to initiate the development of an application that automatically generates the NEWS2 using data from wearables, integrating it with information documented in the Clinical Information System (CIS). Additionally, the study aims to assess the feasibility of this application by evaluating the accuracy of the calculated scores in comparison to scores derived from manually measured vital signs.

## Methods

For this study, we included patients who were hospitalized at the University Hospital of Basel between February and September 2023 and were eligible to participate in the study. Patients were hospitalized in two wards of the department of General Internal Medicine. All patients included were required to provide their written informed consent, which was documented by the participants’ signature. This study was approved by the local cantonal Ethics committee.

Inclusion criteria were age > 18 years and a planned in-hospital-stay of > 24 h. Exclusion criteria were inability to sign written informed consent, significant mental or cognitive impairment as well as medical reasons making it impossible to wear the device, such as allergic reactions, wounds, amputations, excessive hairiness impairing signal quality, edema, installed venous access and others. Baseline data include age, gender, weight and height. Patients with known hypercapnic respiratory failure were analyzed separately to take their lower SpO2-limits (88–92%) into account. Patients skin tone was assessed using Fitzpatrick’s scale [1–6]. Hairiness at the location of the device was graded from 1 – 3 (1 = mild, 2 = moderate, 3 = excessive). In patients with severe body hair, signal quality was checked, and if it led to insufficient data quality, the patients were excluded from further analysis.

For this study, we used the Corsano CardioWatch 287–1, a clinically validated, CE-marked and certified class IIa medical device, which measures heart rate and respiratory rate. More information about the device can be found here: https://corsano.com/wp-content/uploads/2021/04/Corsano-287-1-Leaflet.pdf. Wearable data was collected using the Device Hub IoMT platform developed by the Swiss-based company Leitwert®. Further data was extracted from the clinical information system MEONA®. Due to a missing interface to MEONA we did have to calculate the NEWS2 retrospectively for this trial.

For conventional measurements the Welch Allyn Connex Spot Monitor (Welch Allyn Canada Ltd) was used by nurses. Measurements included an automated oscillometric blood pressure machine using the arm and core temperature using an Ear Thermometer. Oxygen saturation, heart rate and respiratory rate was derived from a Masimo pulse oximetry sensor.

In this trial, while five of the seven parameters required for calculating the NEWS2 score can be measured by wearable devices, none of the currently available devices covered all parameters satisfactorily. Specifically, respiratory rate was measured using wearables, but the accuracy was insufficient for our needs. Although oxygen saturation can be measured by wearables, it was assessed using a separate device (Welch Allyn), as the sensor used in the trial did not capture this parameter. The need for oxygen therapy was recorded manually in the electronic medical record (EMR) by nursing staff when oxygen was provided, eliminating the need for a wearable to track this information. Heart rate was successfully measured with wearable sensors and included in the trial. Although blood pressure can be monitored by wearables, it was measured using a separate device (Welch Allyn) since the trial sensor lacked this capability [12]. Level of consciousness could potentially be integrated into wearables by prompting specific reactions from patients, but this feature is not yet available. Finally, while core body temperature can be tracked by wearables, it was measured with a separate device (Welch Allyn) during the trial due to the wearable sensor’s limitations [13].

### Study procedures

After giving written informed consent, patients were handed a wristband with a PPG-sensor, that was put on the wrist. Participants were instructed to wear the device continuously for three days. Following this timeframe, patients were then asked whether they had worn the device regularly, and if not, to state reasons for that. Furthermore, a semi structured questionnaire was given to ask patients about their experiences with the device. The data collected via the wearable was solely observational and was unavailable to clinical professionals during the study. It had no clinical impact on patient treatment, as all patients received standard medical care.

#### Data collection and analysis

All data collected for the analysis of this study was coded by a unique patient ID. Using a distinct device ID, the wearables were linked to the Bluetooth-to-Wifi gateways, which were integrated with the secure hospital network and controlled by the Device Hub IoMT platform. Raw and processed data was transmitted continuously with Transport Layer Security (TLS) encryption to an in-house database, where it was stored. Under consideration of the typical range of Bluetooth Low Energy connections, the gateways for data transfer needed to be placed in the same rooms as the participants. The Device Hub used in the study allowed to control device status, data availability and signal quality of the wearables. Irregularities, such as poor data quality or missing data were checked regularly by study personnel via the Device Hub user interface. If needed, a check-up was made to detect technical issues or whether the device was not worn by the participants during that time.

At time of patient discharge, NEWS2-Values were calculated and noted in the electronic Case Report Form (eCRF). Data were derived from available measurements in the electronic health record (EHR) and comprised information routinely collected by nurses using standard clinical devices, encompassing all six parameters necessary for NEWS2-calculation. Data were attached with timestamps of measurement.

**Fig. 1:**
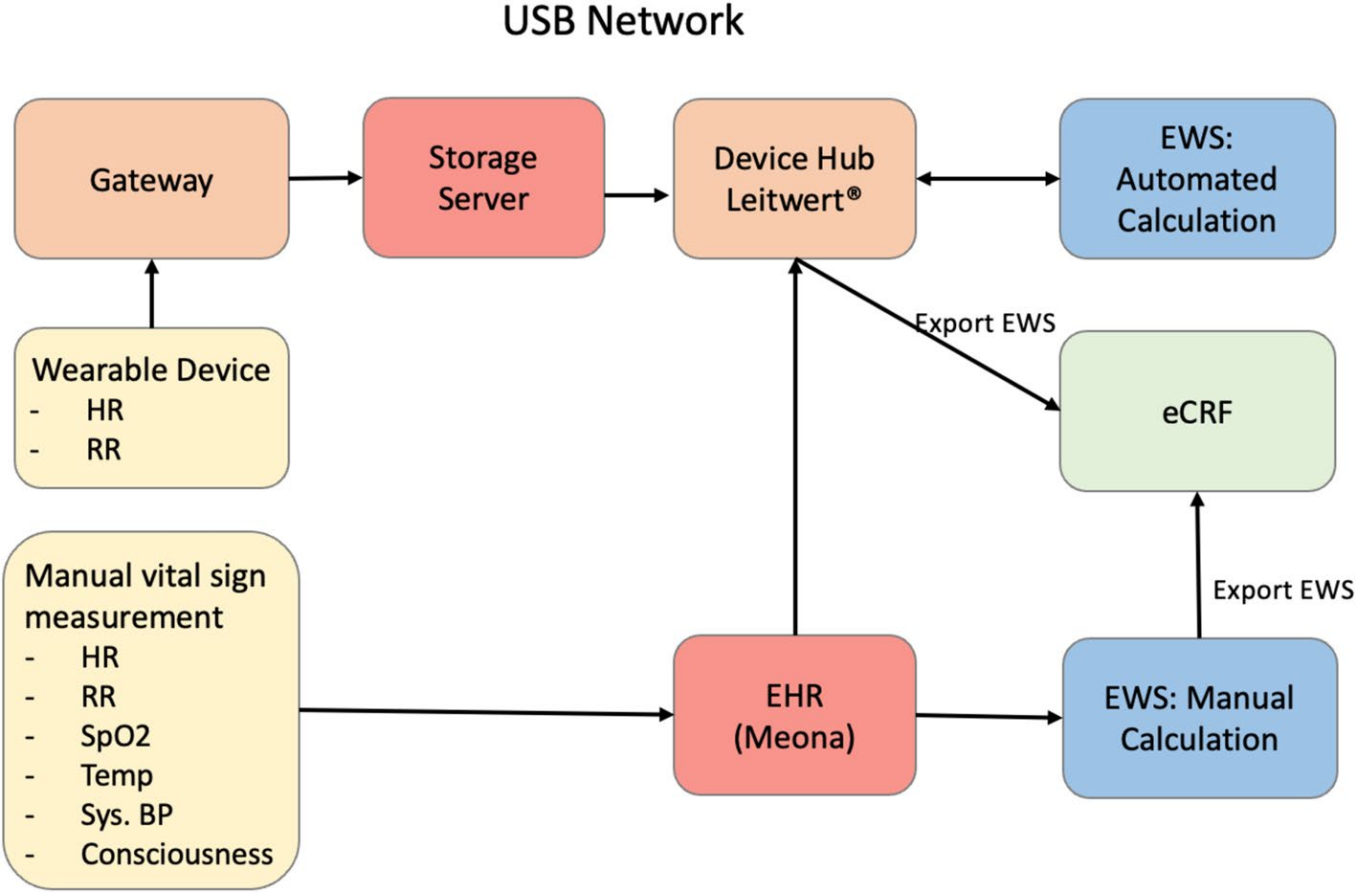
Flowchart showing data measurement and processing. Data acquired by the wearable were combined with conventional measurements stored in the hospitals EHR for further NEWS2-calculation, also conventional measurements stored in the EHR (= Meona) were used for NEWS2-calculation. The final measurements were reported to the eCRF. Abbreviations: eCRF: electronic Case Report Form; EHR: Electronic Health Record; HR: Heart Rate; RR: Respiratory Rate; Temp: Body Temperature; Sys. BP: Systolic blood pressure

Retrospectively, NEWS2 was calculated conventionally with data solely from the EHR and automatically, where NEWS2 was calculated using data from the wearable device (heart rate, respiratory rate) combined with data from EHR (systolic blood pressure, temperature, blood oxygen saturation, consciousness). Data collected by the wearable device was paired with the data documented in the EHR based on timestamps and data quality. To assess for interrater variability between both methods, we used Cohen’s Kappa analysis [14, 15]. Figure 1 details the process of data collection and processing.

If a participant decided to withdraw consent, data which had already been collected was included into the analysis, provided that the participant agreed with its use.

#### Participant questionnaire

After participants had ended the study, a semi structured questionnaire was performed. They were questioned whether they’ve had any prior experience with such wearable devices. Also, they were asked whether they had worn the device regularly and if not, we asked for their reasons. Participants were asked whether they had experienced any difficulties wearing the device and if so, to specify those difficulties. Furthermore, they were questioned about their technical proficiency on a scale between 1 (very good) and 5 (not good at all). Using the same scale, participants were asked on their opinion of continuous monitoring in hospitals, and how safe they would feel if their vital signs were continuously monitored by the device (1 = very safe, 5 = not safe at all). We asked the participants, if they would use such a device at home if it was prescribed to them by their doctor and if not for specific reasons behind it. Lastly, they were asked what they thought potential benefits of wearing such a device could be, but also what concerns they had about such devices. Additionally, we asked them whether they had any further suggestions or comments about the use of such devices in the hospital.

## Statistical analysis

Categorical variables are represented as frequencies and percentages. Continuous variables are presented as mean values ± standard deviation.

NEWS2-measurements collected automatically were graded depending on the respective data quality as determined by the wearable device, with the quality of heart rate and respiratory rate reaching from 1 (low quality) to 4 (perfect quality). For the comparison between conventional and automatic NEWS2, we used only automatic NEWS2 with a data quality of at least 3 for heart rate and respiratory rate. Analysis of the extent of which the automatic NEWS2 matched conventionally measured NEWS2 in a binary classification, meaning critical (≥ 5 points) or non-critical (< 5 points), was performed using Cohen’s kappa, reflecting the agreement rate as a value between 0 (no agreement) and 1 (100% agreement). The cut-offs used in this study were slight agreement (0–0.2), fair (0.21 – 0.40), moderate (0.41–0.60), substantial (0.61–0.80) and almost perfect agreement (0.81–1). The required number of cases for statistical evidence relies sensitively on the frequency of critical cases, with a low frequency requiring more cases. We assumed that 10% of cases would be critical [16, 17]. At the same time, our goal was to show an agreement of at least 0.8 (80%). At a confidence interval of 95%, this resulted in a needed case number of approximately 210. Furthermore, we performed bivariate correlation analysis using Kendall-Tau and Spearman’s correlation tests to analyze for possible correlation between skin type and extent of body hair on data quality. Additionally, we constructed Bland–Altman plots to represent agreement of HR- and RR-measurements. Because HR-measurements did not show normal distribution, tested via Shapiro–Wilk-test and QQ-Plots, the plot for HR-measurements was constructed using the median, upper and lower quantiles. Analysis of the semi-structured questionnaires was performed using qualitative content analysis as proposed by Mayring et.al by summative content analysis [18]. Data analysis was conducted and performed using R Statistics Version 4.3.3. (available at https://www.r-project.org).

## Results

### Baseline characteristics

Of 210 participants, 107 were male (51%), 103 were female (49%). Mean age was 65,9 years (± 16,76). Mean height was 169.7 cm (± 11.88), mean weight was 91.25 kg (± 110.50). Hypercapnic respiratory failure was prevalent in 29 cases (13.8%). 105 participants (50%) had prior known cardiovascular disease. Table 1 gives more details on baseline characteristics; Table 2 presents patients main diagnosis on admission.

**Table 1:** Baseline-Data

Variable	*N* = (%/SD)
**Total**	210
**Male**	107 (51%)
**Female**	103 (49%)
**Age (years)**	65.9 (± 16.7)
**Height (cm)**	169.7 (± 11.8)
**Weight (kg)**	72.5 (± 20.4)
**BMI (KG/m**^2^**)**	25.1 (± 6.3)
**Underweight (BMI < 18.5kg/m**^2^**)**	14 (6.6%
**Normal weighted (BMI 18.5. -24.9kg/m**^2^**)**	106 (50.4%)
**Overweighted (BMI 25.0 – 29.9kg/m**^2^**)**	56 (26.6%)
**Obesity (BMI ≥ 30.0kg/m**^2^**)**	34 (16.2%)
**Hypercapnic respiratory failure**	29 (13.8%)
**Known cardiovascular disease**	105 (50.0%)
**Tatoo in the PPG-sensor area**	2 (1.0%)
**Skin color (Fitzpatrick-Scale)**	
**1**	39 (18.6%)
**2**	114 (54.3%)
**3**	37 (17.6%)
**4**	11 (5.2%)
**5**	5 (2.4%)
**6**	3 (1.4%)
**Hairiness**	
**1 (mild)**	171 (81.4%)
**2 (moderate)**	34 (16.2%)
**3 (excessive)**	5 (2.4%)
**Significant mental/cognitive impairment**	0
**Comprehensible contraindications against smartwatch**	0

**Table 2:** Main diagnosis on admission

Main diagnosis on admission	n (%)
**Infectious**	51 (24.4%)
**Cardiovascular**	33 (15.7%)
**Gastrointestinal**	8 (3.8%)
**Dermatologic**	34 (16.2%)
**Pulmonary**	9 (4.3%)
**Oncologic /hematologic**	6 (2.8%)
**Neurologic**	10 (4.7%)
**Nephrologic/electrolyte disturbance**	4 (1.9%)
**Rheumatologic/Immunologic**	10 (4.8%)
**Endocrinologic**	3 (1.4%)
**Other diagnosis**^**a**^	42 (20.0%)

^a^Main diagnosis on admission of all study participants. “Other diagnosis” includes all patients with diagnoses that were not attributable to any of the above categories. This includes traumatologic, orthopedic, other surgical diagnoses such as neurosurgical diagnoses. Additionally, “Other diagnosis” was picked when the main diagnosis was not clear at the time of screening/study inclusion

### Agreement of automatic and conventional NEWS2

In total, 1511 vital sign measurements were recorded conventionally, with 614 (40.6%) of these measurements being complete to allow NEWS2-calculation. In 444 (29.3%) cases, respiratory rate was the only vital sign missing that disallowed further calculation.

**Fig. 2:**
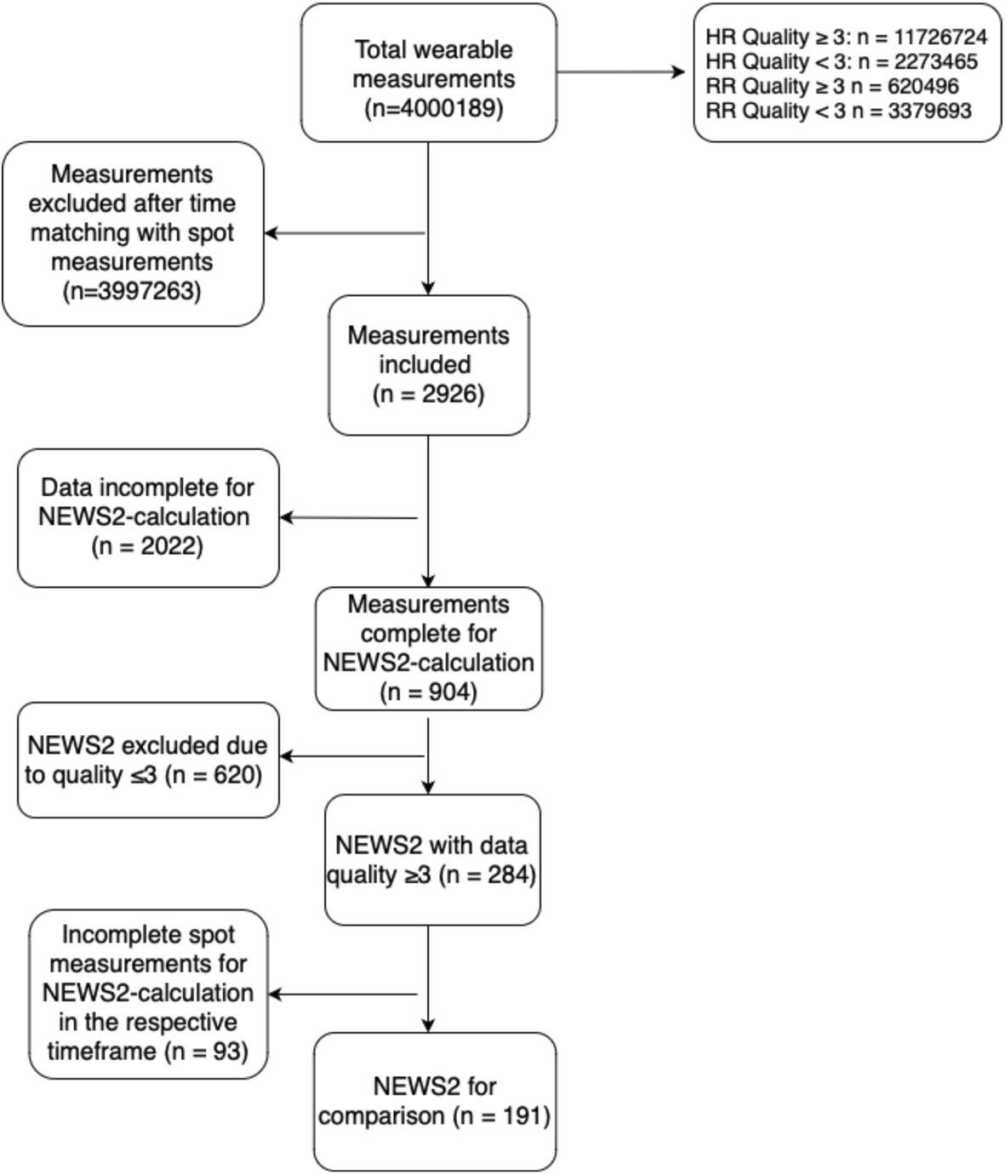
Flowchart of data selection. Abbreviationss: HR: Heart Rate; RR: Respiratory Rate

**Fig. 3:**
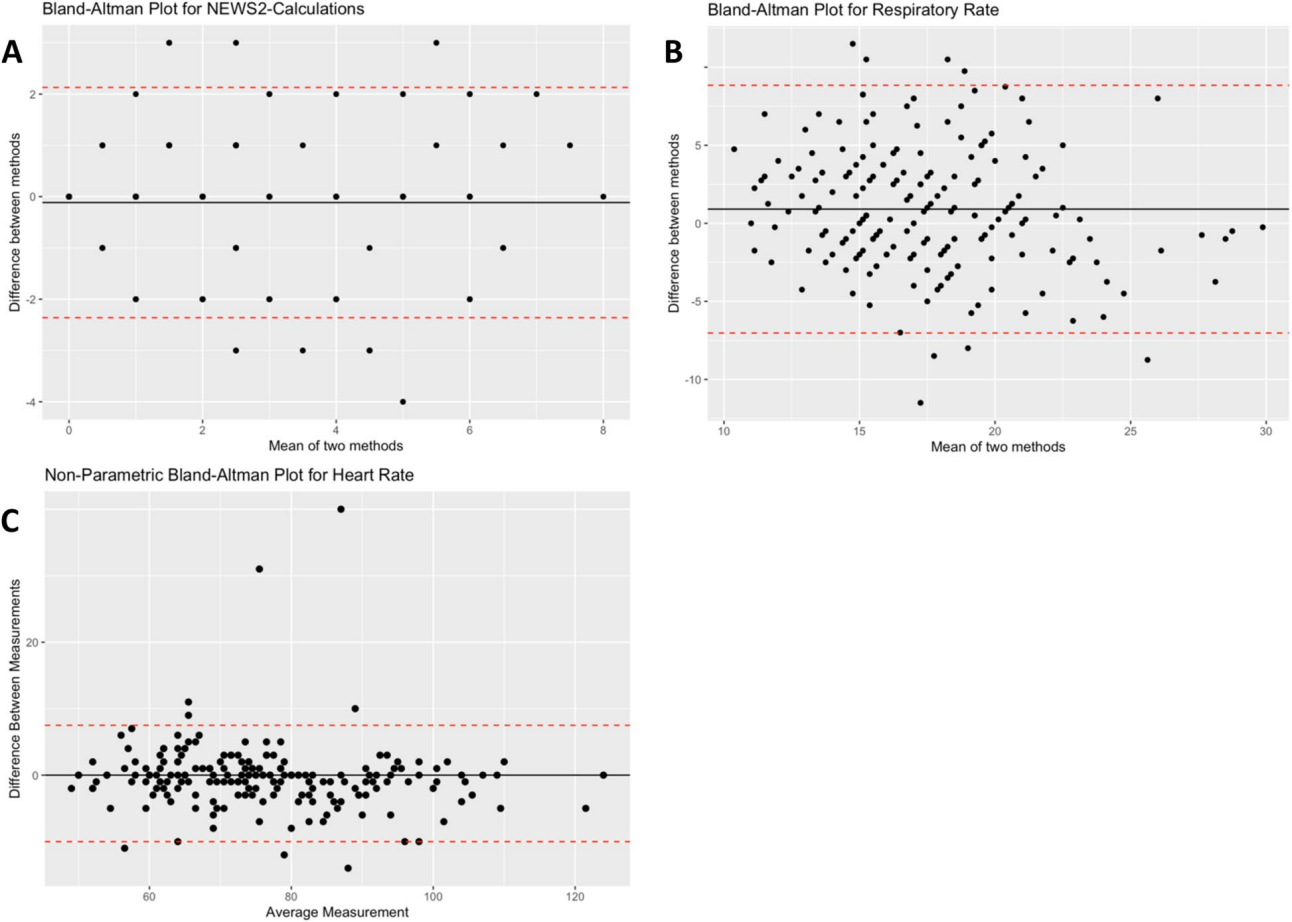
Bland-Altman Plots depicting agreement between NEWS2-calculations (**A**), Respiratory Rate (**B**) and Heart Rate (**C**). The black line shows the mean value of measurements, the red lines show the upper and lower limits of agreement between both measurement methods. Measurements outside of the limits of agreement show insufficient agreement between both methods

Detailed information on data inclusion is presented in Fig. 2. In total, over 4 million vital sign measurements were recorded by wearable devices. The measurements were matched with spot measurements with ± five minutes before or after manual measurements. 2926 wearable cases were available for further analysis. Out of these, we were able to calculate NEWS2 in 904 cases before filtering for data quality. As mentioned above, only data of signal quality ≥ 3 was taken into account for further calculation. After applying this filter, 284 cases were left. Due to insufficient data availability regarding conventional measurements, 191 cases could be used for direct comparison, which were gathered from 100 patients, ranging from one to six measurements. Thirty-three of these measurements resulted in a NEWS2 ≥ 5, 158 in a NEWS2 < 5. Comparing all 191 measurements, accordance was substantial (K = 0.76) between conventional and automated NEWS2. Correlation analysis resulted in a mild positive correlation between body hair extent and data quality of heart rate (R^2^ = 0,064, *p* < 0.001) and respiratory rate (R^2^ = 0.042, *p* = 0.014). There was no correlation between skin type and signal quality. Bland–Altman Plots presenting agreement for NEWS2, HR- and RR-measurements are depicted in Fig. 3. The mean difference for the respiratory rate between the reference monitor and the wireless sensor was + 0.9 breaths/min with wide levels of agreement (95% LoA: − 7.2 to 9.12 breaths/min), for the heart rate + 0.29 beats/min with a 95%CI LoA from − 10.2 to 8 beats/min, respectively. Respective NEWS2 results are shown in Tables 3 and 4.

**Table 3:** Cross-table presenting conventional NEWS2 (x-axis) and automated NEWS2 (y-axis)

NEWS2	0	1	2	3	4	5	6	7	8
**0**	27	3	2	0	0	0	0	0	0
**1**	3	29	0	4	1	0	0	0	0
**2**	0	8	19	4	3	0	0	0	0
**3**	2	0	8	4	0	4	3	1	0
**4**	0	1	2	1	5	1	0	0	0
**5**	0	0	0	1	0	13	0	2	0
**6**	0	0	0	0	1	1	6	2	0
**7**	0	0	0	0	1	2	0	0	0
**8**	0	0	0	0	0	0	1	1	0

**Table 4:** NEWS2-measurements

	**Conventional NEWS2 (*n* = 191)**	**Automated NEWS2 (*n* = 191)**
*0*	32 (19.3%)	33 (19.9%)
*1*	42 (25.3%)	37 (22.3%)
*2*	30 (18.1%)	34 (20.5%)
*3*	14 (8.4%)	22 (13.3%)
*4*	11 (6.6%)	11 (6.6%)
*5*	21 (12.7%)	16 (9.6%)
*6*	10 (6%)	9 (5.4%)
*7*	6 (3.6%)	2 (1.2%)
*8*	0	2 (1.2%)

### Patient questionnaire

The study was terminated irregularly by 58 participants (27.6%). In 2 (3.44%) of these cases, the study was terminated early because the patient’s status declined to a moribund stage. Non-compliance occurred in 2 (3.44%). In one of these cases the patient suddenly refused to wear the device any longer, in the other case the patient’s mental status worsened so the study had to be terminated. Loss of follow up was the most frequent reason and occurred in 45 cases of early study termination (77.5%) because the patients were discharged earlier than expected. No technical problems or cases of product deficiency were registered. Local reaction to the device (itching, slight erythema) was noticed in 1 case and led to termination of the study in this participant. No signs of systemic reaction to the device in form of anaphylaxis were reported. One patient was discharged prematurely (< 24 h), while in 2 cases the patients did not state any reason as to why they did not wear the device per study protocol.

Twenty-seven participants (12.8%) stated that they had not worn the device without interruption. Eight of these participants had taken the device off only temporarily due to a medical examination or intervention, such as MRI, surgery, PET-CT-scan, or they had temporarily been transferred to a different ward where data collection was not possible. Ten participants had not worn the device thoroughly due to local reactions, such as itching at the site of wearable device. Additionally, some participants removed it before taking a shower, possibly due to a misunderstanding as the wearable is waterproof. Five participants had not worn the device regularly due to local injections, lack of space due to bandages or local hematoma.

Difficulties using the device were recorded in 13 cases (6.2%). In most of these cases, participants reported itching of the device, the device being uncomfortable or not fitting right. Two participants reported that the green blinking produced by the wearable device was bothersome.

Overall acceptance of this approach was high. In our survey, 128 patients (60.9%) thought the idea of continuous monitoring in the hospital to be either very good or good. No participants answered the former question with “not good at all”, but 4 participants (1.9%) thought “not good” about the concept. Continuous monitoring led to a higher feeling of safety (very safe or safe) in 94 participants (44.7%), only 1 participant felt “not safe at all”. 130 participants (61.9%) stated that they would use the device even at home if it had been prescribed to them by their doctor. Participants saw multiple benefits in continuous monitoring via our wearable device. The most frequent benefit given was its time efficiency, cost effectiveness and the potential relief on hospital staff. The potential of quicker notification of deterioration of health was seen as highly beneficial, being potentially lifesaving. Patients noted that the device was easy to use. Some participants noted that the opportunity of monitoring even at home could bring some relief on primary care providers. Results of our patient questionnaire are summarized in Table 5.

**Table 5:** Results of the patient questionnaire at the end of the trial

Patient questionnaire	n (%)
**1)** **How tech-savy are you?**	
** Very good**	28 (17.9%)
** Good**	50 (32.1%)
** okay**	32 (20.5%)
** Not good**	24 (15.4%)
** Not good at all**	22 (14.1%)
**2)** **What is your opinion on the idea of continuous Monitoring?**	
** Very good**	60 (38.5%)
** Good**	68 (43.6%)
** okay**	24 (15.4%)
** Not good**	4 (2.6%)
** Not good at all**	0
**3)** **How safe did you feel with your vital signs monitored continuously?**	
** Very safe**	43 (27.6%)
** Safe**	51 (32.7%)
** Okay**	52 (33.3%)
** Not safe**	9 (5.8%)
** Not safe at all**	1 (0.6%)
**4) Would you use this device at home if it was prescribed by your doctor?**	
** Yes**	130 (85.5%)

Regarding potential concerns stated by our participants, concerns about data security were most frequently stated, with potential threats for patients’ privacy. Some individuals expressed concern about an increased reliance on technological devices. Another potential concern reported was lack of interpersonal exchange and relationships may lose importance and space in patient care coming with higher reliance on technical devices. Some participants complained about the device being uncomfortable to wear. Raising wearing comfort was suggested by some participants. Other participants suggested to give the wearer the opportunity to read his own vital signs recorded by the device.

## Discussion

The study found a substantial agreement between automated and conventional NEWS2 calculations from wearable devices. Notably, the automated measurements, drawn from over 4 million data points, closely matched conventional manual measurements. Additionally, patient acceptance of wearable monitoring was generally high, with many participants viewing it as time-efficient and potentially lifesaving, though concerns about data security and comfort were raised.

This prospective study contributes to the growing evidence on continuous monitoring of hospitalized patients using wearable devices. Using the available wearable data to calculate the NEWS2 score, the overall accordance between automated and calculated NEWS2 was substantial. This supports the concept of the usage of wearable devices for continuous monitoring of vital signs in hospitalized patients and the use of an automated NEWS2 Score based on this data. However, this result did not match our pursued goal of a kappa of 0.8 or more. Reasons for this might lie in the low amount of data that were feasible for comparison after filtering for signal quality and matched times of measurements. Out of the analyzed data 68.9% of the heart rate measurement and 30.9% of the respiratory rate measurements were of high quality (quality score ≥ 3). Our Bland–Altman Plots (Fig. 3) showed overall agreement for HR and RR respectively. However, these results need to be interpreted cautiously, as deviations of measurements have different impact on the result of NEWS2-calculation. Above all, the accuracy for RR was outside the limit range we considered acceptable for clinical use. This underlines the present decision to maintain standard monitoring even when wearable sensors are used.

**Fig. 4:**
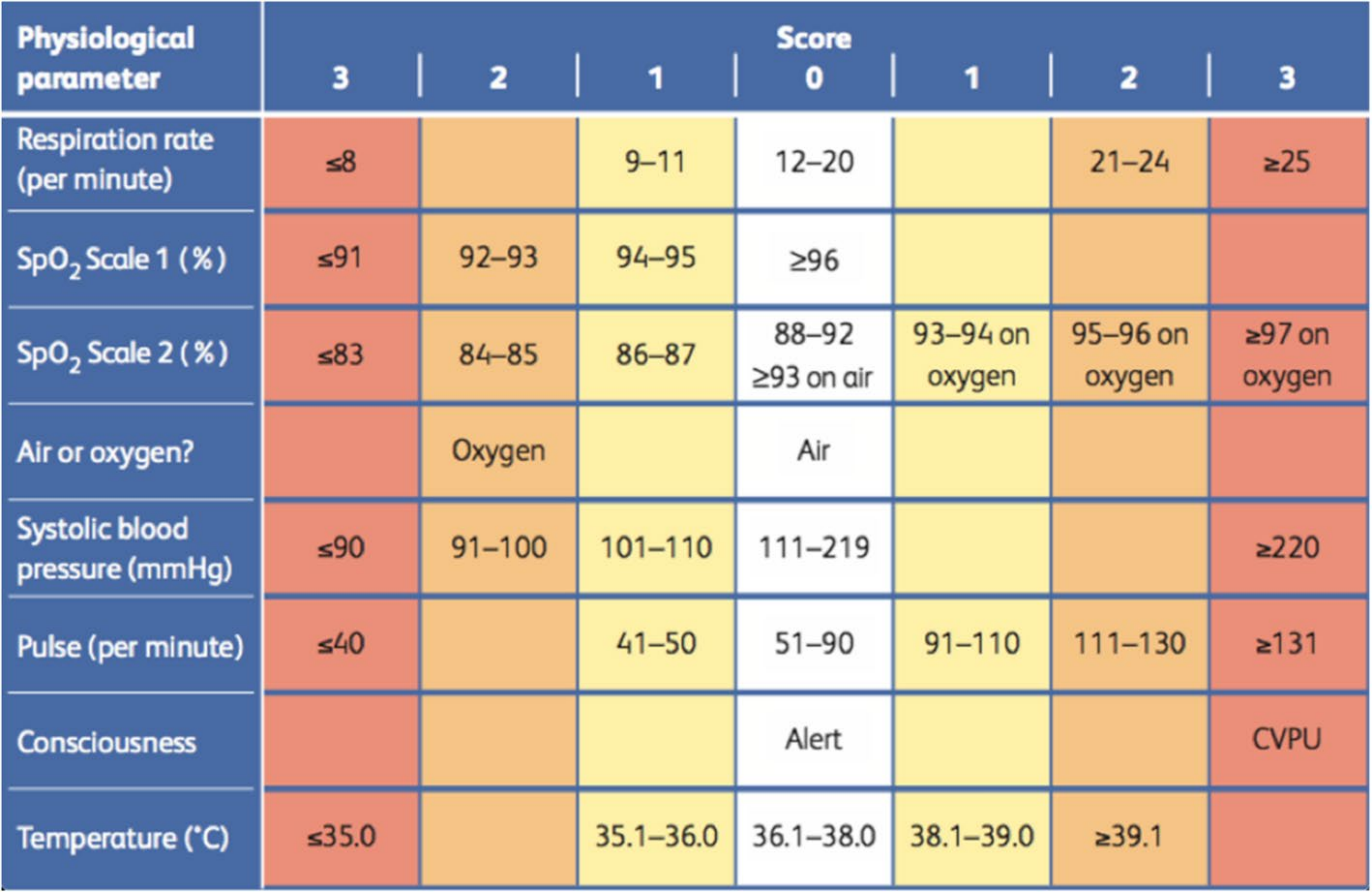
National Early Warning Score 2 (NEWS2) From: Royal College of Physicians. National Early Warning Score (NEWS) 2: Standardising the assessment of acute-illness severity in the NHS. Updated report of a working party. London: RCP, 2017, available at https://www.rcplondon.ac.uk/projects/outputs/national-early-warning-score-news-2

Additionally, as shown in Fig. 4, NEWS2 is more susceptible to differences in RR- as to differences of HR-measurements. For HR-measurements, these deviances could lead to a difference for NEWS2 of 1–2 points, whereas in RR-measurements, deviations yield a higher potential of distortion of NEWS2. For clinical context, this could mean that a patient’s NEWS2 may be significantly over- or underestimated, yielding risks for patient safety. However, this finding may also be due to possible outliers and variability of RR values provided by the reference monitor. Accuracy of the pulse oximetry sensor may be influenced by factors independent of breathing, e.g., movements, coughing and talking. Additionally, the time interval of ± five minutes could have influenced agreement of measurements.

Considering this, a main issue we faced in this study was accordance of respiratory rate-measurements, as depicted in Fig. 3. As mentioned before, respiratory rate is a vital sign often overlooked or ignored by medical staff and its correct measurement is difficult and prone to error [11]. In our clinic, respiratory rate is often only measured using pulse oximetry instead of manual calculation by the nursing staff. In their study, Van Velthoven et al. also found low agreement on respiratory rate measurement [19]. Breteler et al. showed high accordance of heart rate measurements performed with wireless sensors, while the accuracy of respiratory rate measurements was outside of acceptable limits [20]. A reason for the problems of PPG based algorithms to calculate the respiratory rate is, that most, if not all of them include a function of the heartrate variability, which deteriorates in severe medical conditions and makes calculation increasingly unprecise. Additionally, measurements of respiratory rate are often delicate towards external factors [21]. Solving the issue of reliable measurement of respiratory rate will be an important topic for upcoming studies.

Another important point for discussion is how to use wearable devices adequately. The main strength of these devices is the possibility of a continuous measurement. However, in our analysis we only evaluated the measurements of the device, which were recorded five minutes before and after the spot measurement taken by the nursing staff. This could be a strong selection bias, as it resulted in an extreme reduction of usable measurements compared to total available measurements of sufficient quality. Consequently, due to required matching with spot measurements combining manual and wearable measurements with the proposed approach did not improve the availability of NEWS2 scores calculation.

In our study, a considerable number of participants (21.4% of 210 patients) were lost during follow-up. The main reason we reported was early discharge from the hospital. Due to the character of our study, being conducted in a large tertiary hospital, planning of discharge is often difficult and of spontaneous nature, making follow-up for these study settings challenging. Due to a missing interface to our clinical information system, NEWS2 had to be calculated retrospectively instead of the initial goal of calculation via the Device Hub.

General acceptance of this new approach was nevertheless high in our study, with most participants feeling safer under constant monitoring. Analysis of the patient questionnaires shows that overall patients do see the potential benefits of continuous monitoring providing more safety to them and relieving hospital staff, and many participants stated that they would even wear such devices at home if it was prescribed to them. The concerns stated by participants mostly related to safety of patient data, which will be an important topic to manage. As in prior studies, some patients were reluctant to being monitored.

Facing these challenges in accuracy and signal quality we would draw two main conclusions: It was correct to establish this continuous monitoring on top of usual care without influence on medical decisions, and the sensor and the algorithms used to calculate vitals are not sufficient for this kind of task yet.

## Limitations

Our study does have certain limitations. The most important one is that manual measurements of vital signs were performed by different hospital staff, which might have led to errors in data collection. Furthermore, the percentage of respiration rate measurements with sufficient quality by our wearable device was lower than expected. However, other studies have also shown that PPG sensors yield low percentages of respiratory rate measurements with sufficient quality, even though patients in stationary care spend a lot of time at rest. Due to the shorter than planned duration in which the device was worn by participants, we received less data than expected. Because of these circumstances and the study design that requires matching spot and wearable measurements within tight time windows, only 18,5% of conventional measurements and 9.7% of measurements by the wearable device were available for further calculations. Even though loss of follow-up was considerably high with 27.6% not completing the questionnaire, medical data was still recorded by the device as well as manually and was available for calculation of NEWS2 score.

## Conclusions

In conclusion, the study found strong agreement between automated and conventional NEWS2 calculations using wearable devices, with high patient acceptance despite some data quality challenges. To maximize the potential of continuous monitoring, further research into fully automated EWS calculations without relying on spot measurements is suggested, as this could provide a reliable alternative to traditional methods.

## Data Availability

The data and codes relating to the study are available from the corresponding author upon reasonable request.

## Abbreviations

CIS: Clinical Information System
CRF: Case Report Form
eCRF: electronic Case Report Form
EWS: Early Warning Score
HR: Heart Rate
NEWS2: National Early Warning Score 2
RR: Respiratory rate
SpO2: Blood oxygen saturation

## References

[1] Churpek MM, Yuen TC, Edelson DP. Risk stratification of hospitalized patients on the wards. Chest. 2013;143(6):1758–65. 23732586 10.1378/chest.12-1605 PMC3673668

[2] Downey CL, Tahir W, Randell R, Brown JM, Jayne DG. Strengths and limitations of early warning scores: a systematic review and narrative synthesis. Int J Nurs Stud. 2017;76:106–19. 28950188 10.1016/j.ijnurstu.2017.09.003

[3] Alam N, Hobbelink EL, Van Tienhoven AJ, Van De Ven PM, Jansma EP, Nanayakkara PWB. The impact of the use of the Early Warning Score (EWS) on patient outcomes: a systematic review. Resuscitation Mai. 2014;85(5):587–94. 10.1016/j.resuscitation.2014.01.013 24467882

[4] Frank O, Schwappach D, Conen D. Empfehlungen zur Einführung und zum Betreiben eines Früh-warnsystems zur Detektion sich unbemerkt verschlechternder erwachsener Patienten. SAMW. Verfügbar unter: www.samw.ch

[5] Jensen JK, Skår R, Tveit B. The impact of Early Warning Score and Rapid Response Systems on nurses’ competence: An integrative literature review and synthesis. J Clin Nurs. 2018;27(7–8). Verfügbar unter: https://onlinelibrary.wiley.com/doi/10.1111/jocn.14239. Zitiert 15. Nov 2023. 10.1111/jocn.14239 29274170

[6] Welch J, Dean J, Hartin J. Using NEWS2: an essential component of reliable clinical assessment. Clin Med. 2022;22(6):509–13. 10.7861/clinmed.2022-0435 PMC9761428 36427875

[7] Yu SC, Shivakumar N, Betthauser K, Gupta A, Lai AM, Kollef MH, et al. Comparison of early warning scores for sepsis early identification and prediction in the general ward setting. JAMIA Open. 2021;4(3):ooab062. 34820600 10.1093/jamiaopen/ooab062 PMC8607822

[8] Simmes FM, Schoonhoven L, Mintjes J, Fikkers BG, Van Der Hoeven JG. Incidence of cardiac arrests and unexpected deaths in surgical patients before and after implementation of a rapid response system. Ann Intensive Care. 2012;2(1): 20. 22716308 10.1186/2110-5820-2-20 PMC3425134

[9] Van Galen LS, Dijkstra CC, Ludikhuize J, Kramer MHH, Nanayakkara PWB. A protocolised once a day Modified Early Warning Score (MEWS) measurement is an appropriate screening tool for major adverse events in a general hospital population. Lazzeri C, Herausgeber. PLOS ONE. 2016;11(8):e0160811. 27494719 10.1371/journal.pone.0160811 PMC4975404

[10] Petersen JA, Rasmussen LS, Rydahl-Hansen S. Barriers and facilitating factors related to use of early warning score among acute care nurses: a qualitative study. BMC Emerg Med. 2017;17(1):36. 29191159 10.1186/s12873-017-0147-0 PMC5710111

[11] Nicolò A, Massaroni C, Schena E, Sacchetti M. The importance of respiratory rate monitoring: from healthcare to sport and exercise. Sensors. 2020;20(21):6396. 33182463 10.3390/s20216396 PMC7665156

[12] Lee HY, Burkard T. The advent of cuffless mobile device blood pressure measurement: remaining challenges and pitfalls. Korean Circ J. 2022;52(3):198. 35257532 10.4070/kcj.2021.0405 PMC8907988

[13] Etienne S, Oliveras R, Schiboni G, Durrer L, Rochat F, Eib P, et al. Free-living core body temperature monitoring using a wrist-worn sensor after COVID-19 booster vaccination: a pilot study. Biomed Eng Online. 2023;22(1):25. 36915134 10.1186/s12938-023-01081-3 PMC10010220

[14] McHugh ML. Interrater reliability: the kappa statistic. Biochem Med. 2012;22(3):276–82. PMC3900052 23092060

[15] Cohen J. A coefficient of agreement for nominal scales. Educ Psychol Meas. 1960;20(1):37–46.

[16] Lyons PG, Klaus J, McEvoy CA, Westervelt P, Gage BF, Kollef MH. Factors associated with clinical deterioration among patients hospitalized on the wards at a Tertiary Cancer Hospital. J Oncol Pract. 2019;15(8):e652–65. 31306039 10.1200/JOP.18.00765 PMC6694031

[17] Connell CJ, Endacott R, Cooper S. The prevalence and management of deteriorating patients in an Australian emergency department. Australas Emerg Care Juni. 2021;24(2):112–20. 10.1016/j.auec.2020.07.008 32917577

[18] Mayring P. Qualitative content analysis: theoretical foundation, basic procedures and software solution. Klagenfurt. 2014. https://nbn-resolving.org/urn:nbn:de:0168-ssoar-395173.

[19] Van Velthoven MH. ChroniSense national early warning score study: comparison study of a wearable wrist device to measure vital signs in patients who are hospitalized. J Med Internet Res. 2023;25:e40226. 10.2196/40226 PMC9941897 36745491

[20] Breteler MJM, Huizinga E, Van Loon K, Leenen LPH, Dohmen DAJ, Kalkman CJ, et al. Reliability of wireless monitoring using a wearable patch sensor in high-risk surgical patients at a step-down unit in the Netherlands: a clinical validation study. BMJ Open. 2018;8(2):e020162. 29487076 10.1136/bmjopen-2017-020162 PMC5855309

[21] Meredith DJ, Clifton D, Charlton P, Brooks J, Pugh CW, Tarassenko L. Photoplethysmographic derivation of respiratory rate: a review of relevant physiology. J Med Eng Technol März. 2012;36(1):1–7. 10.3109/03091902.2011.638965 22185462

